# 

**Published:** 1878-03

**Authors:** 


### BOOKS AND PAMPHLETS RECEIVED.

Spinal Disease and Spinal Curvature; their Treatment by Suspension and Use of the Plaster of Paris Bandage. By Lewis A. Sayre, M. D., etc. London: Smith, Elder & Co. Philadelphia: J. B. Lippincott & Co. 1878. 12mo.; cloth, pp. 121.

State Regulation of Vice.—Regulation Efforts in America.—The Geneva Congress. By Aaron M. Powell. New York: Wood & Holbrook, publishers. 1878. 16 mo.; cloth, pp. 127.

On the Use of Wines in Health and Disease. By Francis E. Austic, M. D., F. R. C. P.; Late Physician to Westminster Hospital and Editor of the *Practitioner*. Reprint from the *Practitioner*. London: MacMillan & Co. 12 mo.; cloth, pp. 74. Price 75 cents.

Practical Gynæcology. A Handbook of the Diseases of Women. By Heywood Smith, M. A., M. D. Oxon. Member of Royal College of Physicians. Physician to the Hospital for Women and to the British Lying-in Hospital. With illustrations. Philadelphia: Lindsay & Blakiston. 1878. 12 mo.; cloth. Price $1.50.

Handbook of the Practice of Medicine. By M. Charteris, M. D., Prof. of Practical Medicine, Anderson's College, Glasgow, and Physician and Lecturer on Clinical Medicine, Glasgow Royal Infirmary. With illustrations. Philadelphia: Lindsay & Blakiston. 1878. 12 mo.; pp. 336. Price $2.

Landmarks, Medical and Surgical. By Luther Holden, F. R. C. S., etc. From the second English edition. Philadelphia: Henry C. Lea. 1878. 12 mo.; cloth, pp. 128. Price $0.88.

Transactions of the Thirty-second Annual Meeting of the Ohio State Medical Society, held in Put-in-Bay, June 12th, 13th and 14th, 1877. Cincinnati: Mallory & Webb, printers. 1877. 8 vo; cloth, pp. 200.

Nurse, Patient and Camp Cure. By S. Weir Mitchell, M. D. Reprint from Lippincott & Co. 1877. 12 mo.; cloth.

Is the Human Eye Changing its Form Under the Influence of Modern Education? By Edward G. Loring, M. D. New York: 1878.

The Treatment of Fracture of the Femur. By Edward Borck, M. D., etc. Reprint from *St. Louis Medical and Surgical Journal.* 1878.

Truth Admitted. The Columbia Hospital for Women and Lying-in-Asylum. By A Citizen of Washington, D. C. From January number *Richmond and Louisville Medical Journal.*

Seventh Annual Report of the Board of Trustees of the New York Ear Dispensary, incorporated April 8th, 1871. New York: G. P. Putnam's Sons. 1878.

A Report of 15 Cases of Tracheotomy in Diphtheritic Croup, 6 of them Successful. By R. G. Bogue, M. D. Reprint from CHICAGO MEDICAL JOURNAL AND EXAMINER.

Twenty-fifth Annual Report of the Pennsylvania Training School for Feeble Minded Children. Media, Delaware county. 1877.

Report of the Pennsylvania Hospital for the Insane, for the year 1877. By Thomas S. Kirkbride, M. D., Physician-in-Chief and Superintendent. Published by order of Board of Managers. Philadelphia. 1878.

Surgical Treatment of Intra-Uterine Submucous Fibroids. By E. T. Easley, A. M., M. D. From February number *Richmond and Louisville Medical Journal.* 1878

Des Tremblements Consecutifs aux Maladies Aigues. Par Le Dr. E. Clement, Lu à la Société des Sciences Médicales de Lyon. 1877.

Clinical Gynæcology. By W. H. Nathen, M. D., etc. January and February numbers *Richmond and Louisville Medical Journal.*

A Succinct History of the Plan of Treatment of Potts' Disease by Suspension and the Use of Plaster of Paris Bandage. By Lewis A. Sayre, M. D., Prof. Orthopedic Surgery, etc. Reprint from *Richmond and Louisville Medical Journal*, January, 1878.

Circular No. 10. Approved Plans and Specifications for Post Hospitals. Surgeon General's office, Washington, D. C. October 20, 1877.

The Mechanism and Treatment of Pulmonary Complications of Acute Cardiac Disease. By Beverly Johnson, M. D., Physician to Charity Hos pital Reprint from the *Medical Record.*

On Certain Points Relating to the Nature and Treatment of Lupus. By Henry G. Piffard, A. M., M. D., etc. Extracted from Transactions of the Medical Society, State of New York.

Fifty-Second Annual Report of the Massachusetts Charitable Eye and Ear Infirmary, for the year 1871.

Proceedings of the Association of Medical Officers of American Institutions for Idiotic and Feeble-Minded Persons. Sessions, Media, June 6 and 8, 1876; Columbus, June 12 and 15, 1877.

---

DR. VULPIAR writes to the Dean of the London School of Medicine for Women that 33 women have attended the Faculty of Medicine, Paris, since 1865. Of these 6 were English, 12 Russian and 5 French. Of that number 9 have obtained the diploma of doctor.

HARTFORD, Conn., has established a medical journal, and library association. About a thousand volumes have been gathered, mainly books of reference and bound files of journals.

DR. BEVERIDGE, a British naval officer, states that foreign bodies may be removed from the throat by simply blowing forcibly into the ear. This excites coughing by reflex action.

DR. GOWERS has had constructed a modification of Hayem's instrument for counting blood corpuscles, which admits of greater accuracy and can be used with any microscope.

M. RAOUL PISLET has succeeded in obtaining the liquefaction of oxygen gas, under the influence of intense cold and a pressure of 300 atmospheres.

### ANNOUNCEMENTS FOR THE MONTH.

### SOCIETY MEETINGS.

Chicago Medical Society—Mondays, March 4 and 18.

Chicago Society of Physicians and Surgeons—Mondays, March 11 and 25.

### CLINICS.

MONDAY.

Eye and Ear Infirmary—2 to 4 p. m., by Prof. Holmes and Dr. Hotz—2 p. m., Prof. Jones.

Mercy Hospital—2 to 3 p. m. Surgical, by Prof. Andrews.

Rush Medical College—1:30 p. m. Medical, by Dr. Bridge.

County Hospital—8 p. m. Necropsy, by Dr. Danforth.

Woman's Medical College—3 p. m. Surgical, by Prof. Owens.

TUESDAY.

County Hospital—1:30 p. m. Medical, by Prof. Lyman ; 2:30 p. m. Surgical, by Prof. Parkes.

Mercy Hospital—2 p. m. Medical, by Prof. Hollister.

Eye and Ear Infirmary—2 p. m. Prof. Jones.

WEDNESDAY.

County Hospital—1:30 p. m. Ophthalmological, by Dr. Montgomery. 2:30 p. m. Gynecological, by Dr. Bridge.

Mercy Hospital—2 p. m. Eye and Ear, by Prof. Jones.

Rush Medical College—4 p. m. Diseases of the Chest, by Prof. Ross.

THURSDAY.

Mercy Hospital—2 p. m. Medical, by Prof. Davis.

Rush Medical College—1:30 p. m. Neurological, by Prof. Lyman.

Eye and Ear Infirmary—2 to 4 p. m. Operations by Prof. Holmes and Dr. Hotz.

FRIDAY.

Mercy Hospital—2 p. m. Medical, by Prof. Davis.

County Hospital—1:30 p. m. Medical, by Prof. Quine ; 2:30 p. m., Surgical, by Prof. Powell.

Woman's Medical College—10 p. m. Ophthalmological, by Dr. Montgomery.

SATURDAY.

Rush Medical College—2 p. m. Surgical, Prof. Gunn.

Chicago Medical College—2 p. m. Surgical, by Prof. Andrews and Isham ; 3 p. m., Diseases of the Chest, by Prof. Johnson.

Woman's Medical College—12 m. Gynecological, by Prof. Fitch ; 3 p. m. Dermatological, Dr. Maynard.

Special Clinics daily, from 2 to 4 p. m., at the South Side Dispensary, and at the Central Free Dispensary.

For schedule of lectures at the colleges, apply to the college janitors.

